# Comparing Intraoral Radiography and Computed Tomography for Detecting Radiographic Signs of Periodontitis and Endodontic Disease in Dogs: An Agreement Study

**DOI:** 10.3389/fvets.2016.00068

**Published:** 2016-08-31

**Authors:** Robert D. Campbell, Santiago Peralta, Nadine Fiani, Peter V. Scrivani

**Affiliations:** ^1^Department of Clinical Sciences, College of Veterinary Medicine, Cornell University, Ithaca, NY, USA

**Keywords:** periodontitis, endodontic disease, dogs, computed tomography, intraoral radiography

## Abstract

**Objective:**

To determine whether computed tomography (CT) and intraoral radiography are interchangeable for detecting signs of periodontitis and endodontic disease in dogs.

**Materials and methods:**

An agreement study was performed using 40 dogs that previously underwent intraoral radiography and CT during the same anesthetic episode. Images of each tooth were examined by two blinded observers for signs of periodontitis and/or endodontic disease. Agreement between imaging modalities and between observers was assessed using the Kappa statistic.

**Results:**

Agreement between modalities for detecting periodontitis in the maxillae ranged from poor to very good (κ 0.07–1.00) with 16/20 (80%) of the teeth having a score of moderate or better (κ ≥ 0.41). Agreement between modalities for detecting signs consistent with periodontitis in the mandibles ranged from poor to very good (κ 0.01–1.00) with 10/22 (45%) of the teeth having a score of good or better (κ ≥ 0.61); 50% of the disagreement was present in the incisors. Agreement between modalities for detecting signs consistent with endodontic disease in the whole mouth ranged from fair to very good (κ 0.21–1.00) with 30/42 (71%) of the teeth having a score of moderate or better (κ ≥ 0.41). Agreement between observers evaluating intraoral radiology ranged from poor to very good (κ 0.05–1) for detecting signs consistent with periodontitis and from fair to very good (κ 0.36–1) for detecting signs consistent with endodontic disease, in the whole mouth. Agreement between observers evaluating CT ranged from fair to very good (κ 0.35–1) for detecting signs consistent with periodontitis and from fair to very good (κ 0.36–1) for detecting signs consistent with endodontic disease, in the whole mouth.

**Conclusion:**

Performing both CT and intraoral radiography may be unnecessary to detect signs consistent with periodontitis and endodontic disease in dogs based on the amount of agreement between modalities and observers when CT images are acquired and reconstructed in 0.5 or 1 mm slice thickness, except for diagnosing periodontitis in the mandibular incisors.

## Introduction

Intraoral radiology (IOR) has traditionally been the imaging modality used in veterinary dentistry to diagnose dental pathoses (1, 2). Despite its widespread use, IOR has limitations that might underestimate or provide insufficient information to allow clinicians to accurately diagnose disease (3, 4). Common examples of this include superimposition of roots in cases of multi-rooted maxillary dentition, crowding of teeth in brachycephalic breeds, or even inconsistencies caused by subtle variations in the angulation of the X-ray beam. Computed tomography (CT) may overcome some of these limitations due to the production of cross-sectional scans and the ability to perform multiplanar reconstructions using thin slice thickness (5, 6). As CT and cone-beam computed tomography (CBCT) become more available in veterinary hospitals, veterinary practitioners are in the position to diagnose dental diseases based on these images. However, comparison in diagnostic information between IOR and CT scans has not been performed previously.

Two important and common conditions affecting the teeth include periodontitis and endodontic disease. Periodontitis is a plaque-induced chronic inflammatory condition that progressively destroys attachment to the teeth. Left untreated, periodontitis can lead to tooth loss, pathologic jaw fracture, oronasal fistula formation, osteomyelitis, and cellulitis (7, 8). Bone loss secondary to periodontitis has two basic radiographic patterns: vertical and horizontal. Vertical bone loss begins as widening of the periodontal ligament space and continues to progress parallel to the tooth root. Horizontal bone loss occurs parallel to the alveolar margin and is the most common pattern encountered in veterinary patients (9, 10) The amount of attachment loss is an important prognostic indicator for each individual tooth and aids in directing therapy.

Endodontic disease pertains to disease of the vital structures of the tooth (i.e., the pulp). The most common way for pulp to become compromised in the veterinary patient is following tooth fracture or trauma (11). Endodontic disease may lead to localized inflammation and/or infection, and in some cases even result in systemic disease. Therefore, accurate identification and treatment of endodontic disease is important and should not be overlooked (12). Radiographic indicators of endodontic disease include a relatively wide pulp cavity (as compared to neighboring teeth or the contralateral tooth), perapical lysis (apical periodontitis), external inflammatory root resorption, and often obvious loss of crown integrity (13, 14).

In humans, both CT and CBCT have been shown to be superior to IOR for diagnosing periodontal and endodontic disease (15–19). In veterinary medicine, head CT scans are typically performed to diagnose intra- and extracranial disease, nasal disease, or maxillofacial disease not related to the dentition. CT scans performed primarily to diagnose dental disease are uncommon (20). The use of CBCT as a dental imaging modality in dogs has been reported previously (21–23).

Although not previously reported, it is common clinical practice to evaluate the dentition on head CT scans where radiographic signs consistent with periodontitis and endodontic disease can be readily identified. It is unknown how well CT compares to IOR and whether the two techniques may be used interchangeably to support a diagnosis of periodontitis or endodontic disease. If CT is comparable to IOR then IOR may be unnecessary when a head CT scan has already been performed, thereby saving cost and time under general anesthesia by not duplicating diagnostic imaging. Therefore, our study aim was to compare the results of IOR and CT for detecting periodontitis and endodontic disease in dogs with oral, maxillofacial, or dental disease. We hypothesized that on a tooth-by-tooth comparison, that IOR and CT have very good agreement for detecting periodontitis in the mandibular dentition, moderate agreement for detecting periodontitis in the maxillary dentition, moderate agreement for detecting endodontic disease in the whole mouth, and good interobserver agreement for both IOR and CT. The definitions of “very good” and “moderate” will be defined.

## Materials and Methods

The design of this agreement study was retrospective, cross-sectional, and observational. The sample population consisted of all dogs admitted to the Dentistry and Oral Surgery Service at Cornell University Hospital for Animals between September 1, 2013 and May 1, 2015 for evaluation of oral, maxillofacial, or dental disease, and underwent IOR and CT during the same anesthetic event. Exclusions were made for patients with mixed or deciduous dentition. Teeth excluded from evaluation included: teeth involved in a lesion where the periodontium could not be evaluated (i.e., teeth involved in a neoplastic lesion or fracture site); unerupted teeth; and persistent deciduous teeth. In studies with presence of supernumerary teeth, the mesial-most tooth was considered the supernumerary tooth and excluded. Institutional Animal Care and Use Committee approval was exempt for review of the medical records; written client consent was obtained upon admission to the hospital to use their animal’s clinical data for research purposes. The following demographic data were collected for each dog: age, sex, breed, and body weight.

Archived radiographs and CT scans were reviewed by a board-certified veterinary dentist (Santiago Peralta) and third-year veterinary dentistry and oral surgery resident (Robert D. Campbell). The examiners were blinded to each other and to final diagnosis. The order of patient review was randomized for each observer using a series random number generator based on atmospheric noise (www.random.org). CT images were acquired using a 16-slice helical CT scanner^1^ using the same acquisition parameters: 120 kVp, automated mA (SUREExpose – high quality), 0.5 mm slice thickness, pitch factor 1.0, 512 × 512 matrix, and smallest possible scan field of view. From the acquired volumetric CT data, 0.5–1.0 mm CT reconstructions were made in transverse, dorsal, and sagittal planes. All reconstructions were displayed using a bone algorithm. IOR were acquired using a computed radiology phosphor plate system^2^ with radiation exposure times ranging from 0.04 to 0.06 s, with a tube voltage of 60 kV and tube current of 7 mA.^3^ All images were stored and reviewed on a commercially available picture archiving communications system (see text footnote 2).

For IOR, periodontitis was scored for each tooth as “present” when greater than 25% horizontal or vertical bone loss was identified and “absent” when less than 25% alveolar bone loss was present. Endodontic disease was scored for each tooth as “present” when an unexpectedly wide pulp cavity was evident (as compared to neighboring teeth or to the contralateral tooth) and/or if a periapical lucency was present. Endodontic disease was scored as “absent” when pulp cavity width and the periapex appeared normal. For CT, periodontitis was scored for each tooth as “present” when greater than 25% horizontal or vertical bone loss was identified and “absent” when less than 25% alveolar bone loss was present. Detection of periodontitis and endodontic disease by either imaging technique was performed during the same evaluation, meaning that a tooth could be classified as having periodontitis, endodontic disease, both periodontitis and endodontic disease, or be classified as normal.

The statistical methods were selected and performed by two of the authors (Peter V. Scrivani and Robert D. Campbell) using commercially available software^4^ (JMP). Categorical data were described as the frequency of occurrence. Numerical data were assessed for normality using the Kolmogorov–Smirnov test. Normally, distributed data were described using minimum, mean, SD, and maximum. Agreement between IOR and CT was assessed using the Kappa (ĸ) statistic utilizing the following interpretation paradigm: poor (<0.20), fair (0.21–0.40), moderate (0.41–0.60), good (0.61–0.80), and very good (0.81–1.00) (24). Agreement was only assessed when there were two scores (e.g., absent teeth were not counted). For some teeth, there was no instance of periodontitis or endodontic disease detected by both techniques or by both examiners. In this situation, the Kappa statistic was unable to be calculated because it is impossible to divide by 0. The Kappa statistics was scored as 1.00 when there was perfect agreement.

## Results

The sample population consisted of 40 dogs including 6 (15.0%) intact males, 18 (45.0%) neutered males, 2 (5.0%) intact females, and 13 (32.5%) neutered females. The mean age was 7.0 years (SD, 4.2 years; minimum, 0.4 years; maximum, 15.2 years), and the mean body weight was 22.9 kg (SD, 14.9 kg; minimum, 1.2 kg; maximum, 62.5 kg). There were six mixed-breed dogs, three Boxers, three Labrador retrievers, three Yorkshire terriers, two Airedale terriers, two Dachshunds, two German shepherd dogs, two Golden retrievers, two Maltese, two Staffordshire Bull terriers, two Siberian huskies, and one each of the following: American foxhound, Bloodhound, Bulldog, Cocker spaniel, Great Dane, Italian greyhound, Kerry Blue terrier, Newfoundland, Pekingese, Shih Tzu, and Standard schnauzer.

**Figure 1:**
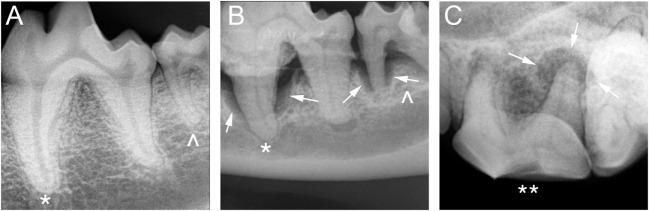
Intraoral radiography: (A) periodontitis and endodontic disease absent in a 4-year-old male-castrated Labrador Retriever in tooth 309 (*) and 310 (^), (B) periodontitis present in a 14-year-old male Dachshund in tooth 309 (*) and 310 (^), and (C) endodontic disease present in a 4-year-old male-castrated Labrador Retriever in tooth 208 (**).

**Figure 2:**
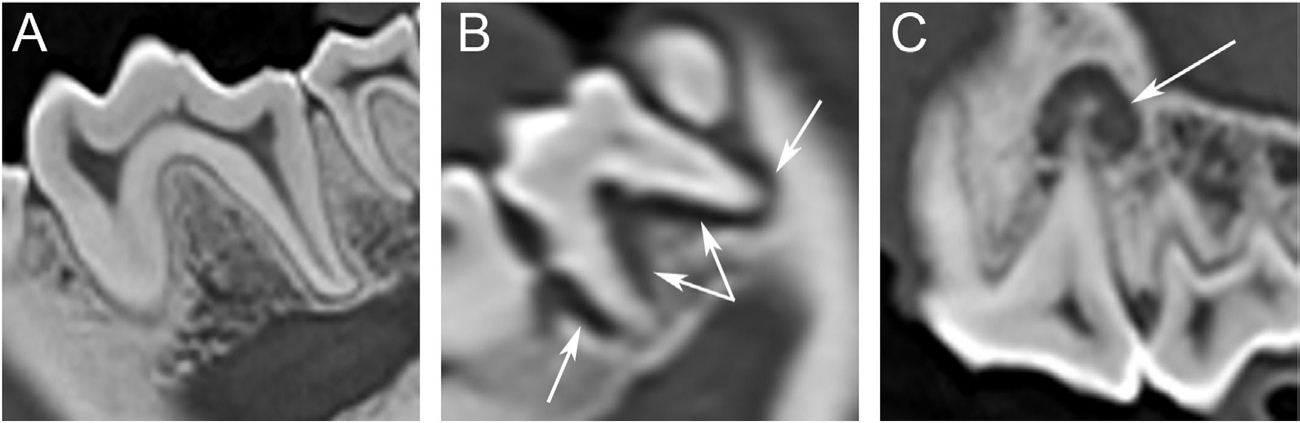
**Computed tomography (CT): (A) periodontitis and endodontic disease absent in a 4-year-old male-castrated Labrador Retriever in tooth 309 and 310, (B) periodontitis present in a 14-year-old male Dachshund in tooth 310, and (C) endodontic disease present in a 4-year-old male-castrated Labrador Retriever in tooth 208**.

All four categories (i.e., periodontitis present, periodontitis absent, endodontic disease present, and endodontic disease absent) were observed in the sample population – but not in each individual tooth – by IOR (Figure 1) and by CT (Figure 2).

### Method Comparisons

Comparisons between IOR and CT for Observer 1 were tabulated by tooth and disease status (Table 1) and summarized as the frequency of observations. For detecting periodontitis in the entire oral cavity, periodontitis was not detected by either technique in 804 teeth; detected by IOR but not CT in 106 teeth; detected by CT but not IOR in 46 teeth; and detected by both techniques in 111 teeth. The frequencies of agreement scores were poor (*n* = 5), fair (7), moderate (12), good (10), and very good (8). For detecting endodontic disease in the entire oral cavity, endodontic disease was not detected by either technique in 994 teeth; detected by IOR but not CT in 25 teeth; detected by CT but not IOR in 29 teeth; and detected by both techniques in 45 teeth. The frequencies of agreement scores were poor (*n* = 3), fair (2), moderate (7), good (13), and very good (12). The Kappa statistic was not determined for five teeth.

**Table 1:** **Method comparison by Observer 1**.

Tooth^a^	Periodontitis					Endodontic disease				
	A	B	C	D	ĸ	A	B	C	D	ĸ
101	22	0	1	4	0.87	26	0	0	2	1.00
102	23	1	1	3	0.71	26	1	1	1	0.46
103	21	2	6	1	0.07	27	0	2	1	0.47
104	25	1	1	4	0.76	30	0	0	1	1.00
105	20	3	1	1	0.25	25	0	0	0	1.00
106	19	1	0	4	0.68	22	0	2	0	NA
107	20	3	1	1	0.25	24	0	0	1	1.00
108	20	1	0	3	0.60	24	0	1	2	0.78
109	20	2	2	3	0.51	25	1	0	2	0.78
110	17	1	3	2	0.40	20	1	2	0	−0.06
201	19	3	1	3	0.51	26	0	0	0	1.00
202	20	3	1	2	0.42	25	0	1	0	NA
203	21	0	4	4	0.59	24	1	3	1	0.27
204	25	0	1	4	0.87	27	1	2	0	−0.05
205	21	3	0	1	0.36	25	0	0	0	1.00
206	20	2	1	2	0.50	23	1	0	1	0.65
207	20	1	2	2	0.50	25	0	0	0	1.00
208	22	0	1	5	0.89	29	1	0	5	0.89
209	19	0	2	3	0.70	21	1	1	1	0.45
210	17	3	1	2	0.40	21	1	1	0	−0.05
301	8	8	0	3	0.24	21	1	0	1	0.65
302	8	11	2	3	0.01	22	0	2	1	0.50
303	15	7	1	3	0.27	25	0	1	1	0.65
304	24	0	0	4	1.00	26	0	1	1	0.65
305	19	3	1	1	0.25	23	1	0	1	0.65
306	19	0	1	1	0.64	20	1	0	1	0.65
307	19	1	0	3	0.83	20	2	1	1	0.50
308	20	1	0	2	0.78	21	1	0	2	0.78
309	21	0	1	5	0.89	23	1	1	2	0.63
310	17	3	0	6	0.72	22	1	0	3	0.84
311	13	3	1	1	0.22	16	0	0	2	1.00
401	18	10	1	5	0.19	23	0	0	1	1.00
402	10	10	4	2	−0.12	30	0	0	2	1.00
403	17	7	1	0	0.07	25	1	0	0	NA
404	24	1	0	3	0.84	26	0	2	0	NA
405	22	1	1	1	0.46	25	1	0	1	0.65
406	26	1	0	1	0.65	26	1	1	1	0.46
407	21	1	0	4	0.67	23	2	1	1	0.34
408	23	0	0	2	1.00	23	1	1	2	0.63
409	20	2	2	3	0.51	24	0	2	1	0.47
410	17	4	0	3	0.52	21	1	0	2	0.78
411	12	2	0	1	0.44	14	1	0	0	NA

*Summary and agreement statistics for IOR and CT by Observer 1 for detecting periodontitis and endodontic disease, by tooth, in all dogs*.

*^a^Modified Triadan system*.

*A = not detected by either IOR or CT*.

*B = detected by IOR but not by CT*.

*C = detected by CT but not by IOR*.

*D = detected by both IOR and CT*.

*ĸ = Kappa statistic*.

For detecting periodontitis in the maxillary dentition, periodontitis was not detected by either technique in 411 teeth; detected by IOR but not CT in 30 teeth; detected by CT but not IOR in 30 teeth; and detected by both techniques in 54 teeth. The frequencies of agreement scores were poor (*n* = 1), fair (3), moderate (8), good (5), and very good (3).

For detecting endodontic disease in the maxillary dentition, endodontic disease was not detected by either technique in 495 teeth; detected by IOR but not CT in 9 teeth; detected by CT but not IOR in 16 teeth; and detected by both techniques in 18 teeth. The frequencies of agreement scores were poor (*n* = 3), fair (1), moderate (3), good (3), and very good (8). The Kappa statistic was not determined for two teeth.

For detecting periodontitis in the mandibular dentition, periodontitis was not detected by either technique in 393 teeth; detected by IOR but not CT in 76 teeth; detected by CT but not IOR in 16 teeth; and detected by both techniques in 57 teeth. The frequencies of agreement scores were poor (*n* = 4), fair (4), moderate (4), good (5), and very good (5).

**Figure 3:**
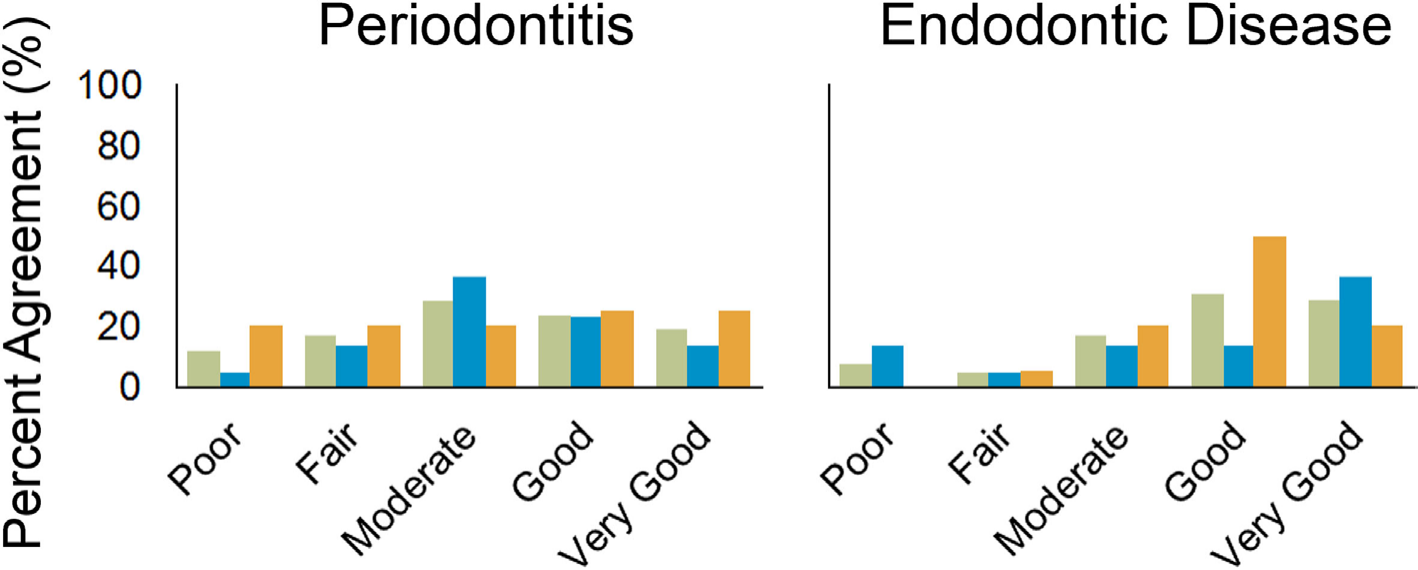
**Bar chart showing percentage of agreement between intraoral radiography and CT by Observer 1 for periodontitis and endodontic disease for the entire oral cavity (green), maxillary dentition (blue), and mandibular dentition (orange)**.

For detecting endodontic disease in the mandibular dentition, endodontic disease was not detected by either technique in 499 teeth; detected by IOR but not CT in 16 teeth; detected by CT but not IOR in 13 teeth; and detected by both techniques in 27 teeth. The frequencies of agreement scores were poor (*n* = 0), fair (1), moderate (4), good (10), and very good (4). The Kappa statistic was not determined for three teeth. These results for Observer 1 also were depicted graphically using the percentages of agreement observations (Figure 3).

Comparisons between IOR and CT for Observer 2 were tabulated by tooth and disease status (Table 2) and summarized as the frequency of observations. For detecting periodontitis in the entire oral cavity by Observer 2, periodontitis was not detected by either technique in 852 teeth; detected by IOR but not CT in 38 teeth; detected by CT but not IOR in 72 teeth; and detected by both techniques in 80 teeth. The frequencies of agreement scores were poor (*n* = 8), fair (6), moderate (7), good (9), and very good (11). The Kappa statistic was not determined for one tooth: in this tooth, periodontitis was not detected by both modalities in 23 dogs and detected by CT but not IOR in 2 dogs. For detecting endodontic disease in the entire oral cavity by Observer 2, endodontic disease was not detected by either technique in 985 teeth; detected by IOR but not CT in 10 teeth; detected by CT but not IOR in 26 teeth; and detected by both techniques in 30 teeth. The frequencies of agreement scores were poor (*n* = 5), fair (1), moderate (2), good (9), and very good (18). The Kappa statistic was not determined for 7 teeth: in these teeth, endodontic disease was not detected by both modalities in 165 teeth, detected by IOR but not CT in 1 tooth, and detected by CT but not IOR in 9 teeth.

**Table 2:** **Method comparison by Observer 2**.

Tooth^a^	Periodontitis					Endodontic disease				
	A	B	C	D	ĸ	A	B	C	D	ĸ
101	21	2	3	0	−0.10	25	0	1	1	0.65
102	22	1	3	1	0.26	27	0	0	1	1.00
103	22	0	5	1	0.24	27	0	1	0	NA
104	25	0	1	3	0.84	29	0	0	0	NA
105	18	2	1	3	0.59	23	1	0	0	NA
106	20	0	1	3	0.83	23	0	1	0	NA
107	22	1	2	1	0.34	25	0	0	1	1.00
108	22	0	1	3	0.83	22	0	1	2	0.78
109	21	1	5	2	0.30	24	1	2	1	0.34
110	20	1	3	1	0.51	24	0	2	0	NA
201	21	2	3	0	−0.10	26	0	0	0	NA
202	23	1	3	0	−0.06	26	0	0	0	NA
203	25	0	1	3	0.84	27	1	1	0	−0.04
204	26	0	0	3	1.00	28	0	0	1	1.00
205	22	1	2	1	0.34	26	0	0	0	NA
206	21	1	1	2	0.62	24	1	0	0	0.00
207	22	1	3	1	0.26	26	0	0	1	1.00
208	22	1	1	2	0.62	22	0	2	2	0.63
209	23	0	2	0	NA	24	0	0	0	NA
210	19	1	3	0	−0.07	20	0	3	0	NA
301	9	4	0	7	0.61	20	0	0	2	1.00
302	14	1	5	1	0.13	20	0	1	1	0.65
303	17	1	3	0	−0.08	21	0	0	0	NA
304	22	1	0	2	0.78	24	0	0	1	1.00
305	21	0	2	1	0.47	23	1	0	0	0.00
306	22	0	0	2	1.00	23	0	0	2	1.00
307	24	0	0	2	1.00	24	1	1	1	0.46
308	21	1	0	2	0.78	23	0	1	1	0.65
309	22	0	0	3	1.00	24	0	1	0	NA
310	19	0	2	4	0.75	22	0	1	2	0.78
311	14	0	1	2	0.77	15	1	0	1	0.64
401	9	5	1	7	0.47	22	0	0	0	NA
402	15	1	6	1	0.10	23	0	0	1	1.00
403	20	1	1	1	0.45	22	0	0	1	1.00
404	24	0	1	2	0.78	24	0	0	2	1.00
405	18	2	3	1	0.17	22	1	1	0	−0.04
406	24	0	0	1	1.00	25	0	0	2	1.00
407	23	1	0	3	0.84	25	1	2	0	−0.05
408	23	0	0	2	1.00	24	0	1	1	0.65
409	22	1	1	1	0.46	24	0	1	0	NA
410	17	2	2	3	0.49	21	1	1	1	0.46
411	15	1	0	2	0.77	16	0	1	1	0.64

*Summary and agreement statistics for IOR and CT by Observer 1 for detecting periodontitis and endodontic disease, by tooth, in all dogs*.

*^a^Modified Triadan system*.

*A = not detected by either intraoral radiography or CT*.

*B = detected by intraoral radiography but not by CT*.

*C = detected by CT but not by intraoral radiography*.

*d = detected by both intraoral radiography and CT*.

*ĸ = Kappa statistic*.

### Observer Comparisons

**Figure 4:**
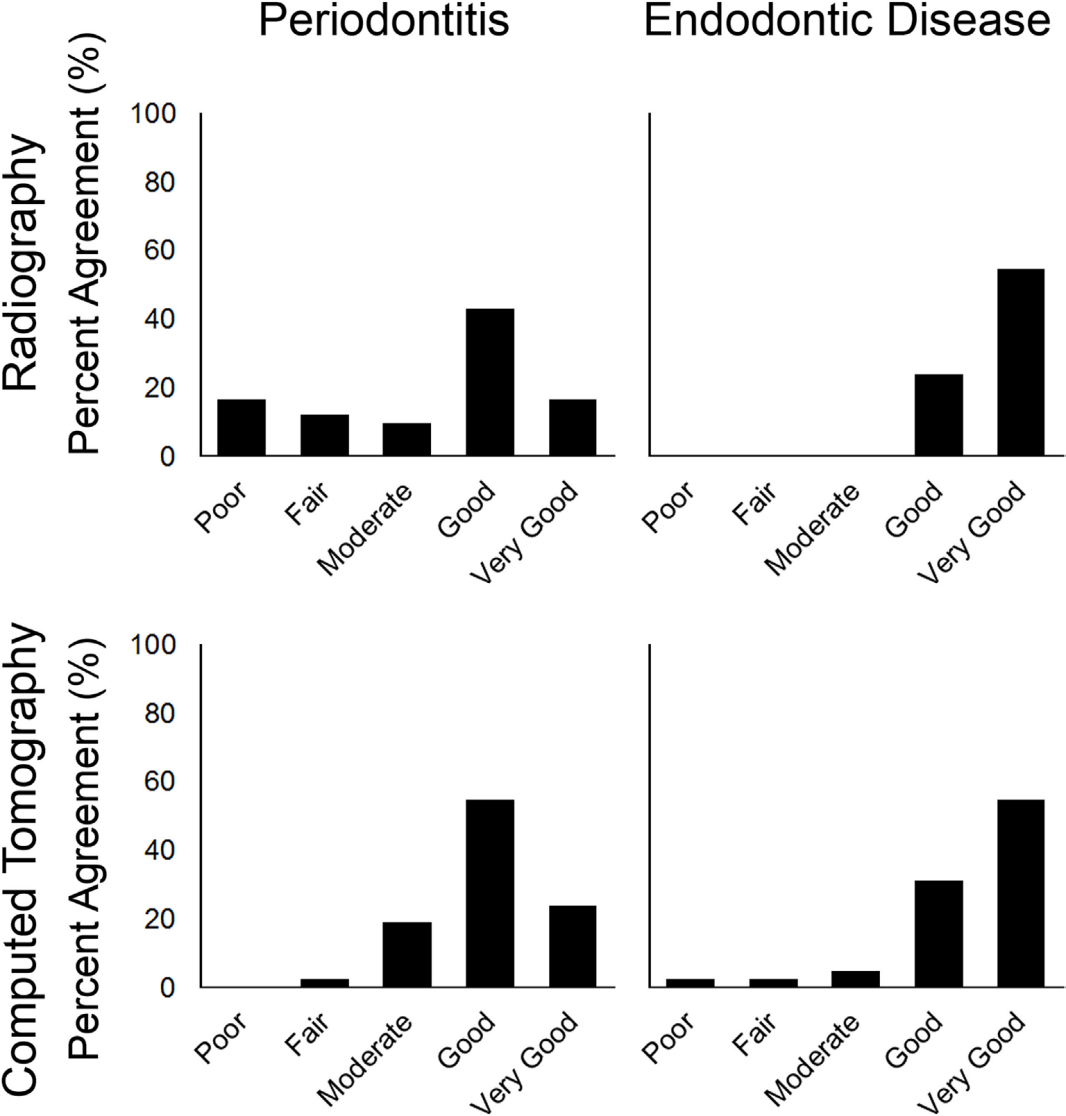
**Bar chart showing percentage of agreement between observers by intraoral radiography and CT for periodontitis and endodontic disease for the entire oral cavity**.

Comparisons between the two observers were tabulated by tooth and disease status for IOR (Table 3) and summarized as the frequency of observations. For detecting periodontitis in the entire oral cavity by IOR: periodontitis was not detected by either observer in 817 teeth, detected by Observer 1 but not Observer 2 in 96 teeth, detected by Observer 2 but not Observer 1 in 23 teeth, and detected by both observers in 112 teeth. The frequencies of agreement scores were poor (*n* = 7), fair (5), moderate (4), good (18), and very good (7). The Kappa statistic was not determined for one tooth. For detecting endodontic in the entire oral cavity by IOR: endodontic disease was not detected by either observer in 977 teeth, detected by Observer 1 but not Observer 2 in 18 teeth, detected by Observer 2 but not Observer 1 in 4 teeth, and detected by both observers in 30 teeth. The frequencies of agreement scores were poor (*n* = 0), fair (0), moderate (0), good (10), and very good (23). The Kappa statistic was not determined for nine teeth. These results also were depicted graphically using the percentages of agreement observations (Figure 4).

**Table 3:** **Observer comparison for IOR**.

Tooth^a^	Periodontitis					Endodontic disease				
	A	B	C	D	ĸ	A	B	C	D	ĸ
101	22	2	1	1	0.34	25	1	0	1	0.65
102	24	1	0	2	0.78	26	1	0	1	0.65
103	27	1	0	1	0.65	29	0	0	0	1.00
104	26	1	0	3	0.84	30	0	0	0	1.00
105	19	1	1	4	0.75	24	0	0	1	1.00
106	19	2	0	3	0.70	24	0	0	0	1.00
107	21	2	0	2	0.63	24	0	0	1	1.00
108	20	2	1	2	0.50	23	0	1	1	0.65
109	19	4	2	1	0.12	24	0	0	2	1.00
110	17	2	3	1	0.16	22	1	0	0	NA
201	24	4	0	1	0.23	25	0	0	0	1.00
202	21	2	0	2	0.63	25	0	0	0	1.00
203	24	1	1	2	0.63	26	1	0	1	0.65
204	26	0	0	3	1.00	28	0	0	1	1.00
205	21	2	0	2	0.63	25	0	0	0	1.00
206	21	1	1	2	0.62	25	0	0	0	1.00
207	21	3	1	1	0.26	25	0	1	0	NA
208	22	1	1	2	0.62	24	0	0	2	1.00
209	21	1	0	0	NA	21	1	0	0	NA
210	17	4	1	0	−0.10	21	1	0	0	NA
301	6	3	1	10	0.59	20	0	0	2	1.00
302	10	10	0	20	0.15	22	0	0	1	1.00
303	16	7	1	1	0.08	25	0	0	0	1.00
304	25	0	0	3	1.00	27	0	0	1	1.00
305	19	4	0	1	0.28	22	1	0	1	0.65
306	20	0	0	2	1.00	20	1	0	1	0.65
307	19	2	0	2	0.62	21	1	0	2	0.78
308	19	1	0	3	0.83	21	2	0	0	NA
309	20	2	1	2	0.50	23	2	0	0	NA
310	16	4	0	4	0.57	21	1	0	2	0.78
311	13	1	0	2	0.76	14	0	0	2	1.00
401	6	4	3	9	0.35	22	0	0	0	1.00
402	13	8	1	1	0.05	23	0	0	1	1.00
403	16	6	1	0	−0.08	23	0	0	1	1.00
404	22	1	0	1	0.65	24	0	1	0	NA
405	20	1	1	2	0.62	22	1	0	1	0.65
406	24	0	0	1	1.00	25	0	1	0	NA
407	19	1	0	3	0.83	22	2	0	0	NA
408	22	1	0	2	0.78	24	1	0	1	0.65
409	22	1	0	2	0.78	25	0	0	0	1.00
410	17	1	1	4	0.74	21	0	0	2	1.00
411	21	1	0	2	0.76	14	0	0	1	1.00

*Summary and agreement statistics for both observers for detecting periodontitis and endodontic disease by IOR, by tooth, in all dogs*.

*^a^Modified Triadan system*.

*A = not detected by either observer*.

*B = detected by first observer but not by second observer*.

*C = detected by second observer but not by first observer*.

*D = detected by both observers*.

*ĸ = Kappa statistic*.

Comparisons between the two observers were tabulated by tooth and disease status for CT (Table 4) and summarized as the frequency of observations. For detecting periodontitis in the entire oral cavity by CT: periodontitis was not detected by either observer in 1135 teeth, detected by Observer 1 but not Observer 2 in 36 teeth, detected by Observer 2 but not Observer 1 in 62 teeth, and detected by both observers in 137 teeth. The frequencies of agreement scores were poor (*n* = 0), fair (1), moderate (8), good (23), and very good (10). For detecting endodontic in the entire oral cavity by CT: endodontic disease was not detected by either observer in 1275 teeth, detected by Observer 1 but not Observer 2 in 12 teeth, detected by Observer 2 but not Observer 1 in 22 teeth, and detected by both observers in 76 teeth. The frequencies of agreement scores were poor (*n* = 1), fair (1), moderate (2), good (13), and very good (23). The Kappa statistic was not determined for two teeth. These results also were depicted graphically using the percentages of agreement observations (Figure 4).

**Table 4:** **Observer comparison for CT**.

Tooth^a^	Periodontitis					Endodontic disease				
	A	B	C	D	ĸ	A	B	C	D	ĸ
101	27	2	2	3	0.53	31	0	1	2	0.78
102	29	1	2	3	0.62	33	1	0	1	0.65
103	25	3	3	4	0.46	31	1	0	2	0.79
104	29	2	1	4	0.68	35	0	0	1	1.00
105	25	0	2	2	0.63	29	0	0	0	1.00
106	25	0	0	5	1.00	28	1	0	1	0.65
107	28	0	1	2	0.78	30	0	0	1	1.00
108	28	1	1	4	0.77	30	0	1	3	0.84
109	29	0	2	5	0.80	33	0	2	1	0.48
110	28	0	1	6	0.91	31	1	1	2	0.63
201	27	1	2	3	0.61	32	0	1	0	NA
202	29	1	1	3	0.72	32	0	0	2	1.00
203	27	3	1	6	0.68	32	0	2	4	0.77
204	30	2	0	4	0.77	33	2	1	0	−0.04
205	29	0	2	1	0.48	32	0	0	0	1.00
206	27	0	1	4	0.87	31	0	0	1	1.00
207	27	1	1	4	0.76	31	0	1	1	0.65
208	29	1	0	5	0.89	29	0	1	5	0.89
209	29	1	1	4	0.77	32	0	0	3	1.00
210	28	1	2	2	0.52	30	0	2	1	0.48
301	19	0	5	4	0.52	26	0	2	2	0.63
302	22	1	3	4	0.59	27	0	0	3	1.00
303	25	0	2	2	0.63	28	0	0	1	1.00
304	27	1	0	3	0.84	29	0	0	2	1.00
305	27	0	1	2	0.78	30	0	0	1	1.00
306	27	1	0	1	0.65	29	0	0	2	1.00
307	29	1	0	2	0.78	31	0	0	3	1.00
308	29	0	0	2	1.00	30	0	0	3	1.00
309	29	2	0	4	0.77	32	1	0	2	0.79
310	26	1	2	5	0.72	30	0	1	3	0.84
311	23	0	2	3	0.71	24	1	1	2	0.63
401	18	1	7	5	0.40	29	0	0	2	1.00
402	20	2	6	4	0.35	30	0	0	2	1.00
403	27	1	1	1	0.46	29	0	1	0	NA
404	29	1	1	3	0.72	32	0	0	3	1.00
405	28	0	2	2	0.64	31	0	1	1	0.65
406	32	0	0	1	1.00	32	0	0	3	1.00
407	31	2	1	4	0.68	34	2	1	1	0.36
408	31	0	0	2	1.00	32	0	0	3	1.00
409	30	2	0	4	0.77	32	2	0	2	0.64
410	28	0	3	3	0.62	30	0	1	3	0.84
411	23	0	0	2	1.00	23	0	1	1	0.65

*Summary and agreement statistics for both observers for detecting periodontitis and endodontic disease by CT, by tooth, in all dogs*.

*^a^Modified Triadan system*.

*A = not detected by either observer*.

*B = detected by first observer but not by second observer*.

*C = detected by second observer but not by first observer*.

*D = detected by both observers*.

*ĸ = Kappa statistic*.

## Discussion

The goal of this study was to compare agreement between IOR and CT in diagnosing radiographic signs consistent with periodontitis and endodontic disease in a group of dogs typical of clinical practice to see if it was necessary to perform both imaging examinations. In humans, CT has been shown to be more accurate than IOR in visualizing experimentally induced periodontal defects (19); CBCT has been suggested to be superior in diagnosing periodontitis and endodontic disease in both humans and dogs (16, 18, 25). To the authors’ knowledge, this study is the first published report of an objective comparison between IOR and CT in clinical veterinary patients evaluated for both signs consistent with periodontitis and/or endodontic disease.

### Periodontitis

The level of agreement between IOR and CT for detecting periodontitis ranged from poor to very good in both the maxilla and the mandible. Interestingly, nearly 50% of the disagreement was due to scoring periodontitis at the mandibular incisors, where periodontitis was detected more frequently with IOR rather than CT. This is likely due to the difficulty when assessing the radiographic appearance of the height of thin alveolar bone in an already crowded location, thereby overestimating periodontitis in these teeth. Conversely, CT may have been better able to identify alveolar margin height and thus have been more accurate in establishing a periodontal diagnosis. Further, interobserver agreement for IOR ranged from poor to very good with 46% of the disagreement present in the mandibular incisors, attesting to the difficulty in differentiating the alveolar margin in this location. The alveolar margin height in the rostral mandible (incisor region) frequently appears radiographically consistent with horizontal bone loss despite no clinical evidence of disease at these teeth (i.e., no clinical attachment loss). These observations are consistent with the findings in this study and indicate the need to further investigate the clinical significance of alveolar margin height at the mandibular incisor area in dogs.

Where disagreements occurred in other teeth in the maxilla and mandible, periodontitis was detected more frequently with IOR as opposed to CT. Experimental studies demonstrating that CBCT imaging is more accurate at diagnosing periodontitis suggests that IOR over diagnoses this disease, and it is reasonable to conclude that CT better delineates alveolar margin height than IOR as well (15, 16, 21, 25).

### Endodontic Disease

Good to very good agreement was identified between CT and IOR for maxillary teeth; the frequency of detection of endodontic lesions was 10% higher for CT as compared to IOR. This discrepancy may be attributed to either under diagnosis of endodontic lesions based on IOR or over diagnosis of lesions based on CT. Although there is no accepted “gold-standard” for dental imaging in veterinary medicine, the preponderance of human and veterinary literature demonstrating that CT and CBCT imaging modalities are superior to IOR for detecting endodontic disease suggests that IOR is underrepresenting the presence of these lesions (18, 26). Moreover, such discrepancy is unsurprising given the complexity of the maxillary dentition and the superimposition of the nasal and periocular structures. Computed tomographic imaging allows for the ability to spatially differentiate anatomy and pathological changes. These findings would support the notion that CT may be indicated when endodontic disease is strongly suspected in the maxilla of dogs but unconfirmed radiographically. Conversely, if endodontic disease is suspected in the mandible, IOR and CT appear to be comparable. This is likely attributed to both less complicated anatomy in the mandible, and the ability to visualize the tooth and periapex without the superimposition present when imaging the maxillary dentition.

Limitations to this retrospective study include the lack of standardization in the image acquisition and reconstruction parameters for CT and lack of standardization in patient positioning and X-ray beam angulation present inherent to IOR. These differences may have made the alveolar margin or periapex difficult to evaluate. Although this limitation may not have allowed for standardized imaging techniques, the variability present in both CT acquisition and radiography is inevitable in the clinical scenario, and therefore may have allowed for a more accurate representation of images generated in practice. CT images in this study were reconstructed in 0.5 or 1 mm slice thicknesses; it is unknown if similar agreement would be present between CT images of differing slice thicknesses (e.g., 2 or 3 mm slices).

Establishing a “gold-standard” imaging technique for periodontitis would allow for future comparison of novel imaging techniques. A prospective cadaveric study with known periodontal and endodontic defects, and standardized imaging parameters, would allow for more impartial scrutiny of the imaging techniques. The absence of a gold standard for dental imaging precluded us from measuring accuracy of CT or IOR and limited the analysis to agreement between the two modalities. Establishing normal radiographic periodontal parameters for the mandibular incisors may provide clinicians with valuable guidance and help prevent over diagnosis of periodontitis in these teeth.

The results of this study support that it is unnecessary to routinely perform IOR following CT (when acquired and reconstructed in 0.5 or 1 mm slice thickness) when testing for endodontic disease in dogs because there is a high level of agreement between techniques and between observers. This recommendation also is true for periodontal disease, except for when evaluating the mandibular incisors. Therefore, we recommend performing radiography following CT only for evaluating periodontitis of the mandibular incisors in dogs. Further, in cases where a head CT has been performed for non-maxillofacial diseases (for example, rhinopathies), the dentition should be thoroughly evaluated and the presence of endodontic and periodontal disease should not be overlooked. It is unknown if varying CT image acquisition parameters would produce comparable agreement between CT and IOR; therefore, if periodontitis or endodontic disease is suspected, clinicians should consider 0.5 or 1.0 mm acquisition and reconstruction slice thicknesses. Images always should be interpreted in conjunction with knowledge of patient data including oral examination. If the results of CT and patient data are in conflict, then performing radiography following CT may be indicated.

## Author Contributions

RC drafted the manuscript, designed the study, and collected and analyzed the data. SP designed the study, collected and analyzed the data, and reviewed the manuscript. NF designed the study and reviewed the manuscript. PS designed the study, analyzed the data, and reviewed the manuscript.

## Conflict of Interest Statement

The authors declare that the research was conducted in the absence of any commercial or financial relationships that could be construed as a potential conflict of interest.

## Footnotes

^1^Aquilion LB, Toshiba American Medical Systems, Tustin, CA, USA.

^2^Carestream, Rochester, NY, USA.

^3^Sirona Dental Systems, Charlotte, NC, USA.

^4^JMP Statistical Software; SAS, Cary, NC, USA.
